# Prospective, multicenter, randomized, controlled trial evaluating the performance of a novel combination powder vs hemostatic matrix in cardiothoracic operations

**DOI:** 10.1111/jocs.14376

**Published:** 2019-11-25

**Authors:** Nicholas C. Dang, Abbas Ardehali, Brian A. Bruckner, Patrick E. Parrino, Daniel L. Gillen, Rachel W. Hoffman, Russell Spotnitz, Stephanie Cavoores, Ian J. Shorn, Roberto J. Manson, William D. Spotnitz

**Affiliations:** ^1^ Department of Surgery Kaiser Moanalua Medical Center Honolulu Hawaii; ^2^ Department of Surgery University of California at Los Angeles Los Angeles California; ^3^ Methodist DeBakey Heart and Vascular Center Houston Methodist Hospital Houston Texas; ^4^ Thoracic and Cardiovascular Surgery Section Ochsner Medical Center New Orleans Louisiana; ^5^ Department of Statistics University of California Irvine California; ^6^ Biom'up SA Lyon France; ^7^ Department of Surgery Duke University Durham North Carolina; ^8^ Department of Mechanical Engineering and Materials Science Duke University Durham North Carolina; ^9^ Department of Surgery University of Virginia Charlottesville Virginia

**Keywords:** bleeding scale, collagen, hemostat, hemostatic agent, hemostatic combination powder, thrombin

## Abstract

**Aim:**

This trial compared the hemostatic performance of a novel combination powder (CP) to a control hemostatic matrix (HM) in cardiothoracic operations.

**Methods:**

Patients meeting eligibility criteria were enrolled after providing informed consent. Subjects were randomized intraoperatively to receive CP (HEMOBLAST Bellows; Biom'up, France) or HM (FLOSEAL Hemostatic Matrix; Baxter Healthcare Corporation, Hayward, CA). Bleeding was assessed using a clinically validated, quantitative bleeding severity scale. The primary endpoint was total time to hemostasis (TTTH), from the start of device preparation, as an indicator of when a surgeon asks for a surgical hemostat until hemostasis was achieved. TTTH at 3 minutes was utilized for the primary analysis, while TTTH at 5 minutes was considered as a secondary endpoint.

**Results:**

A total of 105 subjects were enrolled across four institutions. The primary efficacy endpoint for the superiority of CP relative to HM for success at achieving hemostasis within 3 minutes was met, with 64.2% of the CP group achieving hemostasis compared with 9.6% of the HM group, a difference of 54.54% (37.4%‐71.6%; *P* < .001 for superiority). The secondary efficacy endpoint was also met, with 92.5% of the CP group achieving hemostasis at 5 minutes versus 44.2% in the HM group, a difference of 48.2% (31.1%‐65.4%; *P* < .001 for noninferiority). There were no device‐related adverse events.

**Conclusions:**

In this multicenter, randomized, controlled trial, comparison of CP to HM revealed CP superiority and noninferiority for TTTH at 3 and 5 minutes, respectively.

## 1 INTRODUCTION

Despite advances in surgical techniques, excessive bleeding remains a major complication associated with surgery and contributes to poor clinical outcomes.^1^ Conventional techniques for obtaining hemostasis during surgery include a variety of manual, mechanical, and thermal techniques. Local hemostatic agents may be used in cases where conventional techniques for hemostasis are either ineffective or impractical. Although the properties of the ideal local hemostatic agent may vary according to the surgical specialty, some properties are universally valued including rapid and effective control of bleeding; ability to make effective contact with the bleeding surface; acceptable safety profile; and ease of preparation and use.

The purpose of this study was to evaluate the performance of a recently approved, novel combination powder (CP) compared with an established, control hemostatic matrix (HM) in terms of total time to hemostasis (TTTH; the time from the start of device preparation until hemostasis was achieved). This endpoint captures a time most relevant for surgeons in terms of patient safety and efficiency as it reflects not only effective bleeding control but the ease of preparation as well.

## 2 MATERIALS AND METHODS

### 2.1 Trial design

This was a prospective, multicenter, randomized, controlled trial evaluating the performance of a CP (HEMOBLAST Bellows; Biom'up, France) consisting of collagen, chondroitin sulfate, and thrombin compared to an HM (FLOSEAL Hemostatic Matrix; Baxter Healthcare Corporation, Hayward, CA).

This clinical trial was conducted in accordance with good clinical practice (GCP, ICH E6) and 21 CFR Parts 50, 54, and 56. The trial was registered on ClinicalTrials.gov (NCT #03725098). Institutional Review Board (IRB) approvals and written informed consent from each patient or patient's legally authorized representative were obtained before any study‐specific activities being performed. The date of the first IRB approval was 19 December 2018.

### 2.2 Eligibility criteria

Subjects had to meet all eligibility criteria to be enrolled in the clinical trial. Inclusion criteria were assessed preoperatively and intraoperatively. Preoperative inclusion criteria included: subject undergoing a nonemergent cardiothoracic operation and subject or an authorized legal representative provided prior written consent for trial participation. The intraoperative inclusion criteria included: subject did not have an active or suspected infection at the surgical site; subject in whom the Investigator was able to identify a target bleeding site (TBS) for which any applicable conventional means for hemostasis were ineffective or impractical; and subject had a TBS with minimal, mild, or moderate bleeding, assessed using a clinically validated bleeding severity scale.^2^

Patients were excluded from participation if they had a known sensitivity or allergy to bovine and/or porcine substance(s) or any other component(s) of the hemostatic agents, had religious or other objections to porcine or bovine components or were not appropriate for inclusion in the trial per the medical opinion of the investigator.

### 2.3 Trial endpoints

The primary endpoint of this trial was the superiority of CP relative to HM for the proportion of subjects reaching hemostasis within 3 minutes. The secondary endpoint of this trial was the noninferiority of CP relative to HM for the proportion of subjects reaching hemostasis within 5 minutes, utilizing a noninferiority margin of 10%.

### 2.4 Protocols

Patients were assessed preoperatively to obtain written informed consent for clinical trial participation, confirmation of preoperative eligibility criteria, and collection of demographic data and medical history.

Enrollment and randomization were performed intraoperatively after confirmation of intraoperative eligibility criteria. Randomization occurred immediately following the identification of the TBS. Subjects were randomized to CP or HM in a 1:1 ratio. To ensure balance through time and to maintain concealment of the randomization process, blocked randomization was performed using random block sizes of 2, 4, or 6.

Hemostatic device performance and information regarding the treated TBS were recorded intraoperatively. Patients were discontinued upon completion of the intraoperative visit.

Baseline bleeding severity was assessed using a clinically validated, quantitative surface bleeding severity scale (SBSS).^2^ Minimal, mild, and moderate bleeding severities—corresponding to SBSS (SPOT GRADE) scores of 1, 2, and 3—were eligible for inclusion in study.^2^

TTTH was defined as the time from the start of device preparation, an indicator of when a surgeon asks for a surgical hemostat until hemostasis was achieved. Timing started at the opening of the device packaging and ran continuously until 3 and 5 minutes. Bleeding severity and successful hemostasis were assessed at the 3‐ and 5‐minute time points using the SBSS. Successful hemostasis was defined as no bleeding (an SBSS score of 0). Investigators were trained and tested on the SBSS before the enrollment of subjects.^2^

Additional exploratory outcomes that were assessed intraoperatively included: satisfaction on time and ease of hemostatic device preparation; time of hemostatic device preparation; the incidence of TBS rebleeding; and incidence of device‐related adverse events. Satisfaction was rated on a 5‐point scale using the following definitions: 1 = dissatisfied, 2 = somewhat dissatisfied, 3 = neither satisfied or dissatisfied, 4 = somewhat satisfied, and 5 = satisfied. Both the CP and HM devices were prepared and used according to their approved respective labeling.

### 2.5 Statistical analysis

Continuous endpoints were summarized using descriptive statistics, which included the number of subjects (n), mean and standard deviation. Categorical endpoints were summarized using frequencies and percentages. Two‐sample *t* tests and *χ* ^2^ tests were used to test differences in continuous and discrete baseline covariates between randomized groups, respectively.

The primary efficacy endpoint was defined as the difference in the probability of hemostasis at 3 minutes comparing CP to HM. Letting *θ* denote the true difference in the probability of hemostasis at 3 minutes between CP and HM, the trial tested the null hypothesis *H*_0_: *θ* < 0 vs the alternative hypothesis *H*_a_: *θ* > 0 using a one‐sided level 0.025 two‐sample binomial test of proportions with continuity correction. The corresponding Wald‐based 95% confidence interval (two‐sided) for the difference in probability of the TTTH at 3 minutes was also computed and reported. The secondary endpoint of TTTH within 5 minutes was analyzed in an analogous fashion. Per protocol, exclusion of a 10% difference in favor of HM would signify the noninferiority of CP relative to HM.

Significance testing for the primary and secondary hypotheses was performed in a hierarchical fashion to control the familywise type I error rate of the study. In this case, testing of the secondary endpoint would only have been conducted if statistical significance were met for the primary endpoint. No adjustment for multiple testing was performed for exploratory endpoints. All statistical analyses were performed using Power BI (Microsoft, Redmond, WA) and R.

## 3 RESULTS

### 3.1 Study population

A total of 105 subjects were enrolled and randomized across four investigational sites in the United States consisting of both academic and private practice institutions. Fifty‐three subjects were randomized to the CP group and 52 to the HM group.

The average age of enrolled subjects was 63.7 years and the average body mass index (BMI) was 31.6 (obese). Age and BMI were not significantly different between CP and HM treatment groups (*P* = .323 and *P* = .364, respectively). There was more male than female subjects enrolled, though the distribution of gender was not different between treatment groups (*P* = .120). There were no significant differences in treatment groups in terms of ethnicity and race. Subject demographics are presented in Table 1.

**Table 1:** Baseline demographics for each treatment group

Measure	All	CP	HM	*P* value
Age, y^a^	63.7 ± 11.7 (57.8, 71.2)	62.6 ± 11.4 (57.8, 71.1)	64.9 ± 12.1 (58.0, 73.7)	.323
Gender^b^				.120
Male	81/105 (77.1%)	37/53 (69.8%)	44/52 (84.6%)	
Female	24/105 (22.9%)	16/53 (30.2%)	8/52 (15.4%)	
Ethnicity^b^				.083
Hispanic or Latino	10/105 (9.5%)	8/53 (15.1%)	2/52 (3.8%)	
Not Hispanic or Latino	94/105 (89.5%)	44/53 (83.0%)	50/52 (96.2%)	
Missing	1/105 (1.0%)	1/53 (1.9%)	0 (0.0%)	
Race^b^				.480
Caucasian	49/105 (46.7%)	27/53 (50.9%)	22/52 (42.3%)	
African American	10/105 (9.5%)	4/53 (7.5%)	6/52 (11.5%)	
American Indian or Alaska native	0/105 (0.0%)	0/53 (0.0%)	0/52 (0.0%)	
Asian	24/105 (22.9%)	9/53 (17.0%)	15/52 (28.8%)	
Native Hawaiian or other Pacific Islander	13/105 (12.4%)	7/53 (13.2%)	6/52 (11.5%)	
Other	9/105 (8.6%)	6/53 (11.3%)	3/52 (5.8%)	
BMI, kg/m^2^ ^a^	31.6 ± 30.5 (25.0, 31.6)	28.8 ± 6.6 (24.0, 31.9)	34.3 ± 42.8 (25.1, 30.6)	.364

Abbreviations: CP, combination powder; HM, hemostatic matrix.

^a^ Reported as mean ± standard deviation (p25, p75).

^b^ Reported as n/N (%).

Medical history was similar between treatment groups, with no differences in the rates of concomitant illnesses and preoperative anticoagulation regimen. The most common concomitant illnesses included hypertension, hyperlipidemia, coronary artery disease, heart failure, chronic kidney disease, arrhythmia, myocardial infarction, sleep apnea, chronic obstructive pulmonary disease, and anxiety.

The most frequent surgical procedure for enrolled subjects was coronary artery bypass grafting, followed by valve repair or replacement. As shown in Table 2, the locations of the hemostat‐treated TBS were similar between treatment groups (*P* = .350), with the most common location being the sternum. The dimensions of the TBS were similar between treatment groups (*P* = .321), as were the conventional procedures for hemostasis (*P* = .140).

**Table 2:** Surgical procedure and baseline TBS characteristics for treatment group

Measure	CP	HM	*P* value
Target bleeding site location			.350
Aorta	1/53 (1.9%)	0/52 (0.0%)	
Aortic cannulation site	1/53 (1.9%)	2/52 (3.8%)	
Aortotomy	1/53 (1.9%)	0/52 (0.0%)	
Ascending aorta	6/53 (11.3%)	3/52 (5.8%)	
Left atrium	1/53 (1.9%)	0/52 (0.0%)	
Right atrium	3/53 (5.7%)	2/52 (3.8%)	
Sternum	27/53 (50.9%)	25/52 (48.1%)	
Venous anastomosis site	0/53 (0.0%)	4/52 (7.7%)	
Other	13/53 (24.5%)	16/52 (30.8%)	
TBS approximate dimensions, cm^2^	4.2 ± 6.6	3.1 ± 4.8	.321
	(1.0, 6.0)	(1.0, 2.4)	
Conventional procedures for hemostasis			.140
Cautery	0/53 (0.0%)	2/52 (3.8%)	
Cautery and pressure	4/53 (7.5%)	4/52 (7.7%)	
None practical	34/53 (64.2%)	33/52 (63.5%)	
Pressure	6/53 (11.3%)	2/52 (3.8%)	
Suture	7/53 (13.2%)	11/52 (21.2%)	
Missing	2/53 (3.8%)	0/52 (0.0%)	

Abbreviations: CP, combination powder; HM, hemostatic matrix; TBS, target bleeding site.

The baseline SBSS scores, provided in Table 3, were not significantly different between treatment groups (*P* = .340).

**Table 3:** Baseline SBSS score for each treatment group

SBSS score	All (N = 105)	HEMOBLAST (N = 53)	FLOSEAL (N = 52)	*P* value
0	0 (0%)	0 (0%)	0 (0%)	0.340
1	53 (50.5%)	23 (43.4%)	30 (57.7%)	
2	31 (29.5%)	18 (34.0%)	13 (25%)	
3	21 (20%)	12 (22.6%)	9 (17.3%)	
4	0 (0%)	0 (0%)	0 (0%)	
5	0 (0%)	0 (0%)	0 (0%)	

Abbreviations: SBSS, surface bleeding severity scale.

### 3.2 Primary and secondary endpoints

**Figure 1:**
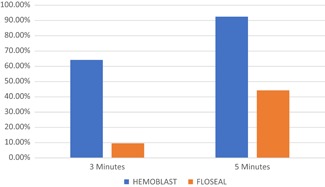
The proportion of subjects in each treatment group achieving hemostasis at 3 and 5 minutes

The proportion of subjects in each treatment group that achieved hemostasis at 3 and 5 minutes is shown below in Table 4 and Figure 1. The CP group had a higher proportion of subjects achieving hemostasis at each time point assessed.

**Table 4:** The proportion of each treatment group achieving hemostasis at 3 and 5 minutes

Time	CP	HM	*P* value
3 min	34/53 (64.2%)	5/52 (9.6%)	<.001
5 min	49/53 (92.5%)	23/52 (44.2%)	<.001

Abbreviations: CP, combination powder; HM, hemostatic matrix.

The primary efficacy endpoint for the superiority of CP relative to HM for success at achieving hemostasis within 3 minutes was met, with 64.2% of the CP group achieving hemostasis at 3 minutes compared to 9.6% of the HM group, a difference of 54.54% (37.4%‐71.6%; *P* < .001 for superiority).

The secondary efficacy endpoint was also met, with 92.5% of the CP group achieving hemostasis vs 44.2% in the HM group, a difference of 48.2% (31.1%‐65.4%; *P* < .001 for noninferiority).

In addition, the proportion of CP vs HM subjects hemostatic at 3 and 5 minutes by baseline bleeding severity (minimal, mild, or moderate) was significantly different, as presented in Table 5 and 6.

**Table 5:** Proportion of subjects hemostatic at 3 min by baseline bleeding severity

Baseline SBSS	HEMOBLAST	FLOSEAL	Difference (95% CI)	*P* value
1 (Minimal)	19/23 (82.6%)	4/30 (13.3%)	69.3%	<.001
	(95% CI, 62.9%; 93.0%)	(95% CI, 5.3%; 29.7%)	(45.7%, 92.8%)	
2 (Mild)	9/18 (50.0%)	1/13 (7.7%)	42.3%	.036
	(95% CI, 29.0%; 71.0%)	(95% CI, 1.4%; 33.3%)	(8.4%, 76.2%)	
3 (Moderate)	6/12 (50%)	0/9 (0.0%)	50%	.043
	(95% CI, 25.4%; 74.6%)	(95% CI, 0.0%; 29.9%)	(12.0%, 88.0%)	

Abbreviations: CI, confidence interval; SBSS, surface bleeding severity scale.

**Table 6:** Proportion of subjects hemostatic at 5 min by baseline bleeding severity

Baseline SBSS	HEMOBLAST	FLOSEAL	Difference (95% CI)	*P* value
1 (Minimal)	22/23 (95.7%)	18/30 (60.0%)	35.7%	.007
	(95% CI, 79.0%; 99.2%)	(95% CI, 42.3%; 75.4%)	(12.4%, 58.9%)	
2 (Mild)	17/18 (94.4%)	3/13 (23.1%)	71.4%	<.001
	(95% CI, 74.2%; 99.0%)	(95% CI, 8.2%; 50.3%)	(39.5%, 100%)	
3 (Moderate)	10/12 (83.3%)	2/9 (22.2%)	61.1%	.019
	(95% CI, 55.2%; 95.3%)	(95% CI, 6.3%; 54.7%)	(17.0%, 100%)	

Abbreviations: CI, confidence interval; SBSS, surface bleeding severity scale.

### 3.3 Exploratory outcomes

The average satisfaction ranking was 4.8 ± 0.6 for the CP group, compared with 3.5 ± 1.5 for the HM group, which was statistically significant (*P* < .001). The mean preparation times were 19.5 ± 9.8 seconds for CP and 2 minutes and 26.4 ± 52.3 seconds for HM (*P* < .001).

### 3.4 Safety

There were no reported device‐related adverse events in either treatment group. However, there was a significant difference in the incidence of rebleeding between treatment groups, with no cases in the CP group and 11 cases in the HM group (*P* = .001).

## 4 DISCUSSION

Hemorrhage control is vital for successful clinical outcomes after surgery. It is essential to decrease postoperative morbidity and operative time, leading to potential cost savings.^3^, ^4^, ^5^ During surgical operations, it is important to maintain the fine balance between bleeding and clotting so that blood continues to flow to the tissues at the surgical site without excessive blood loss. There are many tools used for hemorrhage control; these include preventive measures, transfusion of blood products, and conventional methods, as well as local hemostats.^1^ The ideal hemostat, sealant, or adhesive must have certain performance characteristics (safety, efficacy, usability, cost, and approvability) that enable it to be used by surgeons.^6^

CP is a novel active powdered hemostat consisting of collagen, chondroitin sulfate, and thrombin. It has demonstrated safety and efficacy in a pivotal clinical trial.^7^ Its safety and efficacy are further confirmed in this trial compared with a control HM flowable agent, where success was measured using TTTH. The powdered hemostat is available for use in under 20 seconds, compared with 2 minutes and 26 seconds for the HM. The mean satisfaction score of 4.8 for the CP device indicates overall surgeon satisfaction, compared with a mean satisfaction score of 3.5 for HM, which indicates less than somewhat satisfied. The preparation time and satisfaction scoring for CP are indicative of CP's degree of usability. Additionally, the cost of CP is competitive with other hemostatic devices. It is currently approved by the United States Food and Drug Administration and is CE marked. The data collected in this trial, as well as another prospective, randomized, controlled trial with CP, provide evidence that CP has many of the characteristics of the ideal hemostat.^6^, ^7^

This trial utilized TTTH that measures the time starting when bleeding requiring a local hemostat is identified and stopping when hemostasis is achieved. This is the actual time it takes for a surgeon to wait for hemostasis to be achieved, and therefore is a highly clinically relevant measurement of time to hemostasis and a measure of the safety of using the hemostat. The TTTH may be extended when using agents that require thawing, reconstitution, mixing, and/or further steps of preparation. Flowables and fibrin sealants have reported preparation times of approximately 3 minutes up to 30 minutes. This preparation time may require advance reconstitution of these products at the start of the surgical procedure with the possible waste of unused product or reconstitution after a bleeding site is identified causing delay of application of the product and thereby possibly exposing the patient to additional time of bleeding.^7^, ^8^, ^9^, ^10^

There is no preparation time for CP as it is essentially immediately available. The difference between the mean preparation times for CP and HM in this study was over 2 minutes and was significantly different. Using a combination powder such as CP may result in decreased operating room time, eliminate the need for operating room staff to prepare the hemostat, reduce operating room waste by avoiding the need for any preprepared product, enhance the safety of the patient by reducing delays in achieving hemostasis, and potentially save cost. Additional studies have examined time to hemostasis and included preparation or application times^11^ as well as the importance of hemostatic agent selection in influencing clinical outcomes and treatment costs.^12^ Operating room time and waste are becoming more prevalent concerns.^13^ Product costs contribute to the increasing costs of medical care and include the cost of time to use the product.^14^, ^15^ The cost of operating room time has been reported to range from $62 to $133 per minute.^10^, ^16^, ^17^ Therefore, time spent to prepare hemostatic agents can contribute to overall surgical procedure costs and should be considered when making a decision on which hemostatic devices to use. In addition to evaluating the initial purchase cost of hemostatic products, it is apparent that consideration should be given to the cost of time required to prepare and apply them; the value of a product may be determined by calculating its time‐cost efficiency.^10^

Though there were no reported device complications or device‐related adverse events in this study, rebleeding of a treated target bleeding was noted at the HM and not at the CP site. Rebleeding can be seen as both a performance and a safety issue. Rebleeding requires the surgeon to revisit the TBS to address the bleeding. Furthermore, if rebleeding goes unnoticed during the primary surgical procedure, it could result in poorer postoperative outcomes or even require reoperation, additionally increasing the risk to the patient and overall treatment cost.

### 4.1 Strengths and limitations

A limitation of this trial was the lack of double‐blinding. Investigators were blinded as to the treatment modality until after the TBS was identified and assigned a bleeding severity. However, due to the physical differences between the two devices, investigators were not blinded after subject randomization to the treatment group and the time of product application.

## 5 CONCLUSION

In this multicenter, randomized, controlled trial, CP compared with HM showed superiority and noninferiority for TTTH at 3 and 5 minutes, respectively, in cardiothoracic surgery using a validated, quantitative bleeding severity scale. In addition, CP had a significantly shorter preparation time and higher ratings for usability when compared with HM.

## CONFLICT OF INTERESTS

Ardehali is a member of the Board of Directors of Biom'up SA. Bruckner and Gillen are paid consultants of Biom'up SA. Hoffman, R. Spotnitz, Cavoores, Schorn, Manson, and W. Spotnitz are employees of Biom'up SA.

## AUTHOR CONTRIBUTIONS

RWH, RS, SC, IJS, and WDS conceived and designed the study. DLG and RWH conducted data analysis and interpretation. RWH drafted the article. DLG, RJM, and WDS critically revised the article. WDS approved the article. DLG and RWH conducted the statistical analysis. NCD, AA, BAB, and PEP collected data..
